# Antioxidant Effects of PS5, a Peptidomimetic of Suppressor of Cytokine Signaling 1, in Experimental Atherosclerosis

**DOI:** 10.3390/antiox9080754

**Published:** 2020-08-14

**Authors:** Sara La Manna, Laura Lopez-Sanz, Susana Bernal, Luna Jimenez-Castilla, Ignacio Prieto, Giancarlo Morelli, Carmen Gomez-Guerrero, Daniela Marasco

**Affiliations:** 1 Department of Pharmacy, CIRPEB: Centro Interuniversitario di Ricerca sui Peptidi Bioattivi- University of Naples “Federico II”, 80134 Naples, Italy; sara.lamanna@unina.it (S.L.M.); giancarlo.morelli@unina.it (G.M.); 2 Renal and Vascular Inflammation Group, Instituto de Investigacion Sanitaria-Fundacion Jimenez Diaz (IIS-FJD), Autonoma University of Madrid (UAM), 28040 Madrid, Spain; laura.lsanz@quironsalud.es (L.L.-S.); susana.bernal@quironsalud.es (S.B.); luna.jimenez@quironsalud.es (L.J.-C.); ignacio.prieto@quironsalud.es (I.P.); 3 Spanish Biomedical Research Centre in Diabetes and Associated Metabolic Disorders (CIBERDEM), 28040 Madrid, Spain

**Keywords:** mimetic peptides, cytokine signaling, JAK/STAT, suppressor of cytokine signaling 1 (SOCS1), oxidative stress, atherosclerosis

## Abstract

The chronic activation of the Janus kinase/signal transducer and activator of the transcription (JAK/STAT) pathway is linked to oxidative stress, inflammation and cell proliferation. Suppressors of cytokine signaling (SOCS) proteins negatively regulate the JAK/STAT, and SOCS1 possesses a small kinase inhibitory region (KIR) involved in the inhibition of JAK kinases. Several studies showed that KIR-SOCS1 mimetics can be considered valuable therapeutics in several disorders (e.g., diabetes, neurological disorders and atherosclerosis). Herein, we investigated the antioxidant and atheroprotective effects of PS5, a peptidomimetic of KIR-SOCS1, both in vitro (vascular smooth muscle cells and macrophages) and in vivo (atherosclerosis mouse model) by analyzing gene expression, intracellular O_2_^•−^ production and atheroma plaque progression and composition. PS5 was revealed to be able to attenuate NADPH oxidase (NOX1 and NOX4) and pro-inflammatory gene expression, to upregulate antioxidant genes and to reduce atheroma plaque size, lipid content and monocyte/macrophage accumulation. These findings confirm that KIR-SOCS1-based drugs could be excellent antioxidant agents to contrast atherosclerosis.

## 1. Introduction

Atherosclerosis is a chronic, systemic vascular inflammatory disease, and it is responsible for at least 50% of all deaths in the Western world [1]. Generally, it is characterized by endothelial dysfunction, lipid deposition in the artery wall and excessive, unresolved inflammation [2]. Oxidative stress-induced vascular injury also represents a major contributor to the pathoetiology of atherosclerosis. Indeed, an imbalance between the activity of pro-oxidant systems (e.g., NADPH oxidase (NOX), mitochondrial respiratory chain, lipoxygenase and xanthine oxidase) and antioxidant enzymes (e.g., superoxide dismutase (SOD), glutathione peroxidase, catalase (CAT), thioredoxin peroxidase, paraoxonase and heme oxygenase), leading to increased generation of reactive oxygen species (ROS), is implicated in the initiation and progression of atherosclerosis [3].

Common cardiovascular risk factors such as hypertension, diabetes mellitus, hypercholesterolemia and smoking enhance ROS levels, which can oxidize different lipoproteins, limit the vascular availability of antiatherosclerotic nitric oxide and promote vascular expression of cytokines and adhesion molecules [4]. The major source of ROS in vascular cells is represented by NOX enzymes that catalyze the production of superoxide from oxygen and NADPH, a coenzyme widely involved in a broad range of redox reactions that regulate reduced glutathione levels [5]. The excessive production of NOX-dependent ROS is linked to atherogenesis causing vascular dysfunctions, defective angiogenesis and immune-inflammatory activation. In the vasculature, NOX1, NOX2 and NOX4 are the isoforms predominantly expressed by different cell types in the intima, media and adventitia layers and may contribute to oxidative stress in both human and experimental atherosclerosis [6]. Stimuli promoting sustained activation of vascular NOX include pro-inflammatory cytokines (e.g., interleukin (IL)-6 and interferon (IFN)-γ), growth factors, peptide hormones, mechanical forces, metabolic intermediates, modified lipids and lipoproteins [7]. Based on this evidence, selective NOX inhibitors might be novel therapeutics to prevent and/or treat cardiovascular diseases [8].

The activation of Janus kinase/signal transducers and activators of the transcription (JAK/STAT) pathway is mainly constituted by the formation/disassembly of protein complexes and strictly correlated with the expression of inflammatory genes during the early stages of atherogenesis including the leukocyte recruitment, the migration and proliferation of vascular smooth muscle cells (VSMCs), the apoptosis and the formation of lipid-laden macrophages and VSMCs or foam cells [9,10,11,12,13,14]. Evidence exists that JAK2 and STAT1/3 are crucial regulators of NOX expression and activity in cultured VSMCs and atherosclerotic mice [15,16,17]. The JAK/STAT pathway is controlled by suppressor of cytokine signaling (SOCS) proteins with a negative feedback regulatory mechanism. The inhibition of the main family members—SOCS1 and SOCS3—leads to sustained cytokine activation of STATs and contributes to the progression of inflammatory (including cardiovascular) diseases [18]. During the early phases of atherogenesis, the activation of STAT3 promotes the differentiation, migration and proliferation of VSMCs and the formation of neointima after vascular injury. In detail, STAT3 activation induces a decrease in SOCS3 levels leading to increased resistin, cytokine expression and intracellular ROS production dependent on NADPH oxidase (by increased NOX1 expression) [19,20]. On the other hand, SOCS1 protein shows both pro-apoptotic and anti-apoptotic functions. Indeed, during the ROS-mediated apoptosis of immune cells, SOCS1 expression is induced by ROS, but conversely, SOCS1 overexpression leads to the inhibition of oxidant-induced apoptosis [21,22]. In experimental models, both gene deficiency and pharmacological inhibition of JAK2, STAT1 and STAT3 avoided atherosclerotic lesion formation [23,24]. SOCS-based strategies to impair pathological JAK/STAT activity were already reported for the treatment of cardiovascular diseases confirming the anti-inflammatory and atheroprotective properties of the SOCS1/SOCS3 gene delivery in experimental atherosclerosis models [25,26]. These studies demonstrated that SOCS1 is able to block ROS generation by inhibiting NOX because of the upstream inactivation of JAK2, STAT1 and phosphoinositide 3-kinase (PI3K) [12,25,27]. The SOCS family is formed by eight proteins sharing a similar organization of domains constituted by an N-terminal region of variable length and sequence, a central SH2 domain and a C-terminal SOCS box [28]. Only SOCS1 and 3 present a kinase inhibitory region (KIR) [29], and several studies of therapeutic potential of compounds based on KIR sequence outlined their promising properties in vitro and in inflammatory animal models [30,31,32,33]. In addition, our recent reports on mouse models of diabetes demonstrated relevant anti-inflammatory and antioxidant features of the peptide corresponding to the KIR fragment, placing it as a starting compound for the design of compounds to limit the progression of chronic complications of diabetes [26,34].

**Figure 1:**
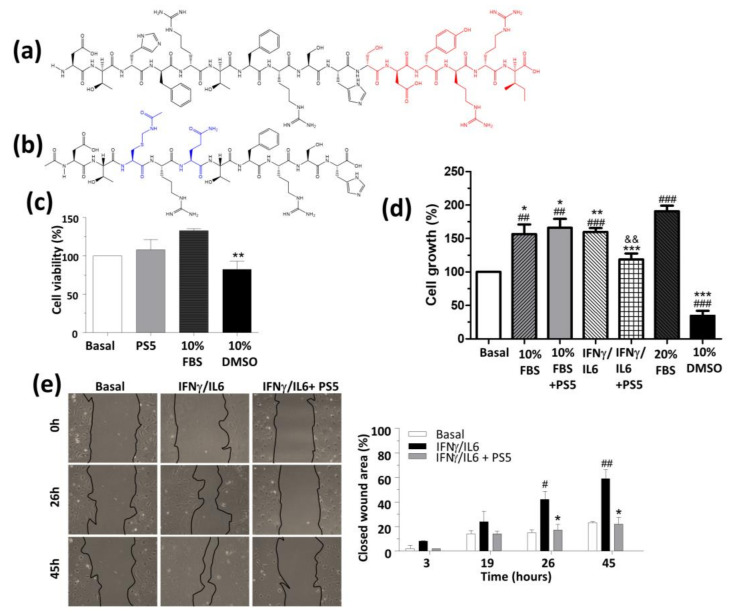
Structure and in vitro effects of PS5 peptide on vascular smooth muscle cells (VSMCs). Chemical structure of (**a**) KIR SOCS1 and (**b**) PS5 peptide (red, residues deleted by Ala-scan analysis; blue, non-native residues). (**c**) MTT assays to evaluate cell viability in VSMCs after 6 h of incubation in a medium containing 0.5% FBS and 25 µM PS5 peptide, using 10% FBS and 10% DMSO as positive and negative controls, respectively. (**d**) VSMC proliferation assay in medium containing 10% FBS or IFNg/IL6 in the absence or presence of 25 µM PS5 (20% FBS and 10% DMSO as positive and negative controls). (**e**) Scratch wound healing assay in VSMCs. Representative images and quantifications of covered healing areas at the indicated times, expressed as a percentage of the initial wound area. Results are presented as the mean ± SEM of *n* = 2–3 independent experiments. ^#^ *p* < 0.05, ^##^ *p* < 0.01 and ^###^ *p* < 0.001 vs. basal; * *p* < 0.05, ** *p* < 0.01 and *** *p* < 0.001 vs. respective positive control; and *p* < 0.01 vs. IFNγ/IL6.

To identify new therapeutics endowed with anti-inflammatory/antioxidant properties, the use of peptide-based compounds mimicking greater proteins is experiencing considerable interest in recent decades [35,36,37,38,39,40,41]. For example, several studies pointed out that Thioredoxin-1 is an antioxidative, anti-inflammatory protein with atheroprotective effects, and recently, a thioredoxin-mimetic peptide showed anti-oxidative, anti-inflammatory and atheroprotective effects in ApoE2.Ki mice [42]. Similarly, the apolipoprotein (apo) A-I mimetic compound 5A was revealed to be able to confer in vivo protection against inflammation and oxidation through specific interaction with ATP-binding cassette transporter A1 [43]. On this basis, we developed a peptidomimetic of KIR SOCS1, named PS5, that was identified through the screening of focused combinatorial peptide libraries [44,45]. Using an alanine-scanning approach, the peptide encompassing residues 52–67, i.e., KIR of SOCS1 was reduced to a shorter fragment including only amino acids crucial for the interaction with JAK2, region 52–61 and deleting residues 62–67 evidenced in red in Figure 1a. Subsequently, ”non-essential” amino acids located in the N-term part of KIR were substituted by distinct amino acids following a simplified combinatorial approach. The related screening in positional scanning format provided PS5 as a lead compound that bears an un-natural residue Cys (-Acm), at position 54, reported in blue along with the other non-native residue Gln at position 56 (Figure 1b). PS5 was revealed to be able to bind to JAK2 with K_D_ values in the nanomolar range [45,46] and was tested in cultures of human keratinocytes and murine VSMCs. In these cell types, we demonstrated a direct effect of PS5 on JAK/STAT since PS5 was revealed to be able to reduce the phosphorylation of STAT1 and STAT3 and the expression levels of interferon regulatory factor-1 (IRF-1) and caused a strong reduction in the expression of inflammatory genes such as intercellular adhesion molecule-1 (ICAM-1), human leukocyte antigen-DR isotype (HLA-DR), C-X-C motif chemokine 10 (CXCL10), C-C motif chemokine ligand 2 (CCL2) and CCL5 [46,47]. Here, to evaluate the possibility to extend the therapeutic potential of PS5 to cardiovascular diseases, we investigated its antioxidant and atheroprotective effects both in vitro, in VSMCs and macrophages, and in vivo, in a mouse model of atherosclerosis.

## 2. Materials and Methods

### 2.1. Peptide Synthesis

Both PS5 and negative control (NC) peptides were synthesized on a 50 μmol scale following standard Fmoc procedures of SPPS, as already reported [45]. The molecular weight of the peptides and the purity of fractions were evaluated by LC-MS analysis. To perform cellular assays, both peptides were linked to the fragment 48–60 of the HIV Tat protein at the N-terminus of the peptides and labelled with carboxytetramethylrhodamine (TAMRA) fluorophore. Purified peptides were lyophilized, then dissolved in a water solution at 1 mM stock concentration, filter-sterilized and stored aliquoted at −20 °C under N_2_ until further use. Reagents for peptide synthesis were from Iris Biotech (Germany); reversed phase columns for peptide analysis and the LC-MS system were from ThermoFisher (Waltham, MA); solvents for peptide synthesis and HPLC were from Romil (Dublin, Ireland).

### 2.2. Cell Cultures

Primary VSMCs from mouse aorta were isolated by enzymatic digestion with collagenase type II and cultured in Dulbecco’s modified Eagle medium (DMEM) supplemented with 10% fetal bovine serum (FBS), 100 U/mL penicillin, 100 µg/mL streptomycin and 2 mM L-glutamine (Sigma-Aldrich), and used between the 4th and 7th passages [12]. VSMCs were characterized by immunostaining with smooth muscle actin (positive) and factor VIII (negative) and by real-time PCR analysis of differentiation markers of contractile phenotype (actin alpha 2 (*Acta2*) and smooth muscle protein 22-alpha (*Sm22a*)) and synthetic phenotype (Kruppel-like factor 4 (*Klf4*) and matrix metalloproteinases (*Mmp2* and *Mmp9*)).

Mouse macrophage cell lines RAW 264.7 (TIB-71, ATCC) were cultured in DMEM with 10% FBS, 100 U/mL penicillin, 100 µg/mL streptomycin and 2 mM L-glutamine (Sigma-Aldrich, Milan, Italy). Quiescent cells (24 h in a medium with 0.5% FBS (VSMC) or 0% FBS (RAW 264.7)) were pretreated with PS5 and NC peptides conjugated to TAT derived cell penetrating sequence (25 μM, 90 min) before stimulation with mouse recombinant cytokines (IFNγ 10^3^ U/mL plus IL-6 10^2^ U/mL; PeproTech, London, U.K.) for 6 h.

### 2.3. Cell Viability, Proliferation and Migration

Both cell viability and proliferation were measured by MTT colorimetric assay [48,49]. For cell viability, VSMCs were plated in 96-well plates (1 × 10^4^ cells/well) and allowed to attach overnight at 37 °C, then incubated overnight in serum-free media at 37 °C. PS5 and NC peptides were then added to the plate (25 µM) and incubated for an additional 6 h in DMEM containing 0.5% FBS, using a medium with 10% FBS as the positive control. An MTT solution was then added for 1 h. The absorbance of the metabolized MTT was measured at *λ* = 570 nm in a plate reader. For the cell proliferation assay, VSMCs (5 × 10^3^ cells/well) were incubated for 24 h in DMEM containing 10% FBS or cytokines (IFNγ 10^3^ U/mL plus IL-6 10^2^ U/mL) in the presence or absence of peptides, using 20% FBS as the positive control. Cells were then processed similarly to viability assay. In both assays, a medium with 10% DMSO was used as the negative control.

The migratory capacity of VSMCs was measured by wound-healing assay [25]. VSMCs were plated in 12-well plates (3 × 10^5^ cells/well) and depleted for 24 h in a medium with 0.5% FBS; a wound injury was performed using a sterile 200-μL pipette tip, and then cells were incubated with cytokines in the presence or absence of peptides (25 µM). To determine the closing speed of the wound, two images of each well were captured at different stimulation times (0, 3, 19, 26 and 45 h). Wound closure was quantified (Image Pro-Plus; Media Cybernetics) and normalized with respect to the initial time (t = 0).

### 2.4. Superoxide Measurement by DHE

VSMCs and RAW 264.7 cells on glass coverslips were incubated with the fluorescent dye dihydroethidium (DHE) at 2.5 µM in KRB-HEPES buffer (10  mM HEPES pH 7.4, 1.2  mM MgCl_2_, 2.5  mM CaCl_2_, 119  mM NaCl, 4.7 mM KCl, 1.2  mM KH_2_PO_4_, 25  mM NaHCO_3_ and 2  mM glucose) for 30 min at 37 °C. After several washes, to remove excess of fluorescent probe, cells were pretreated with peptides (25 µM, 90 min) and stimulated for 3 h with cytokines (IFNγ plus IL-6). Then, cells were nuclear counterstained with 4′,6-diamidino-2-phenylindole (DAPI) and mounted. The samples were analyzed by confocal fluorescence microscopy (λexc = 488 nm and λem = 570–600 nm). ROS levels were expressed as a percentage of DHE positive cells versus total cells (DAPI nuclear staining).

### 2.5. Mouse Model of Atherosclerosis and Treatments

The housing and care of animals and all the procedures carried out in this study were strictly in accordance with the Directive 2010/63/EU of the European Parliament and were approved by the Institutional Animal Care and Use Committee of IIS-Fundacion Jimenez Diaz and Comunidad de Madrid (PROEX 116/16 date of approval 26.05.2016; PROEX 217/19 date of approval 21.08.2019). Male Apolipoprotein E knockout (ApoE KO) mice aged 8–10 weeks were fed a high-fat diet (Western type TD88137, 20–23% fat, 0.2% cholesterol; Harlan Teklad) and administered PS5 peptide by intraperitoneal injection 3 days/week for 8 weeks. Mice were randomly divided into four groups: (i) untreated control (*n* = 8), (ii) treated with low-dose PS5 (19 µg/mouse; *n* = 4), (iii) treated with medium-dose PS5 (38.1 µg/mouse; *n* = 8) and (iii) treated with high-dose PS5 (79.4 µg/mouse; *n* = 8). Animal weights were monitored weekly for the entire duration of the in vivo experiment to ensure that the dose differences were doubled (from low to medium and from medium to high dose groups, respectively). At the end of the study, 16 h fasted mice were anesthetized (100 mg/kg ketamine and 15 mg/kg xylazine), saline-perfused and killed. The dissected aorta was divided into two parts: the upper aortic root was embedded in optimal cutting temperature medium (OCT) and cryo-sectioned for histology; the thoracoabdominal aorta was processed for RNA isolation.

For biodistribution experiments, mice received a single intraperitoneal injection of TAMRA-PS5 peptide. At 6 and 24 h post injection, the in vivo and ex vivo imaging was performed using an IVIS Lumina (Caliper Life Sciences) coupled with Living Image software (Xenogen Corporation).

### 2.6. Histological Analysis and Quantification

To analyze the area and composition of atheroma plaques, sequential 7 µm cross-sections (covering ~1000 µm from valve leaflets) were stained with Oil-red-O/hematoxylin. Macrophages were detected in parallel sections by immunohistochemistry using an anti-CD68 primary antibody (ab53444, Abcam) followed by a biotinylated secondary antibody (anti-rat, 712-035-150; Jackson ImmunoResearch), avidin-biotin complex reagent and 3,3′-diaminobenzidine substrate (Vector Laboratories). The atherosclerotic lesion area (µm^2^) and the content of neutral lipids and macrophages were quantified using Image-Pro Plus software (Media Cybernetics). The individual lesion area was determined by averaging the maximal values (2–3 sections). Positive staining was reported as a percentage of the total area.

### 2.7. mRNA Expression Analysis

Tryzol reagent (Life Technologies) was used to extract total RNA from cultured cells and mouse aorta. Target gene expression (*Nox1*, *Nox4*, *Sod1*, *Cat*, *Ccl2*, *Ccl5*, *Cxcl10*) was analyzed in duplicate by quantitative real-time PCR (Applied Biosystem), and mRNA values were normalized to the housekeeping gene 18S rRNA; the primers for PCR detection are listed in Table 1.

### 2.8. Statistical Analyses

Results are shown as individual values—the mean ± standard error of the mean (SEM) of at least 3 independent experiments. Statistical analysis was performed using Prism 5 (GraphPad Software Inc), and *p*-value < 0.05 was considered significant (one- or two-way ANOVA with Bonferroni’s posthoc test).

## 3. Results

### 3.1. In Vitro Effects of PS5 Compound

#### 3.1.1. Cellular Viability, Proliferation and Migration

To perform cellular experiments, the PS5 sequence (Figure 1b) was covalently attached to the cell-penetrating peptide covering the fragment 48–60 of the HIV Tat, and the ability of PS5 to interfere with cell viability and proliferation was investigated through MTT colorimetric assay. As shown in Figure 1c, PS5 demonstrated no cytotoxic effects on cell viability at 25 μM. Interestingly, PS5 did not affect VSMC proliferation after 24 h of incubation in a culture medium with 10% FBS but was able to suppress the mitogenic effect of the combination of pro-inflammatory cytokines IFNγ/IL6 (Figure 1d). Because VSMC migration and proliferation are essential features of vascular remodeling during atheroma plaque formation [50], we further performed in vitro wound-healing assay to assess the activity of PS5 peptide on collective cell migration. As already reported for KIR peptide [34], PS5 significantly diminished the migration and proliferation (“wound healing”) capacity of VSMCs by the quantifications of healed areas that exhibit a lower percentage of cell-covered area after PS5 pretreatment (Figure 1e). Indeed, while pro-inflammatory cytokines IFNγ/IL6 promoted a time-dependent increase in VSMC migration, PS5 exerted a significant, sustained anti-migratory effect over 45 h, which could be due to a combination of proliferation and migration inhibition.

#### 3.1.2. Cellular ROS Production

**Figure 2:**
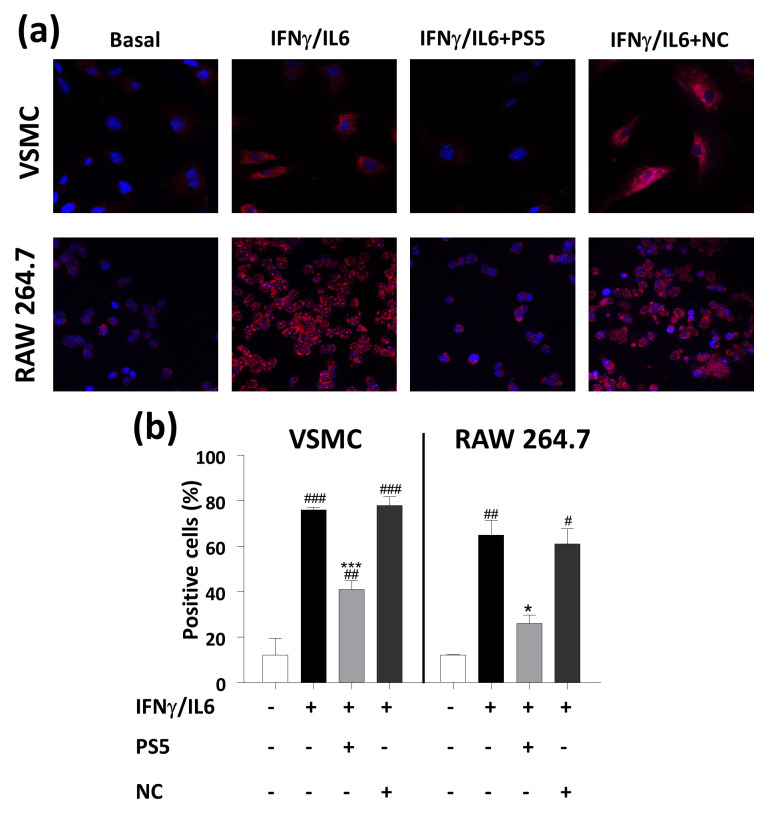
PS5 effects on ROS production. (**a**) Representative confocal images of intracellular O_2_^•−^ (red, DHE staining; blue, DAPI nuclear staining) in VSMCs and RAW 264.7 cells stimulated with cytokines in the presence or absence of PS5 and NC peptides. (**b**) Quantification of PS5 activity based on DHE fluorescence intensity. Results are presented as the mean ± SEM of *n* = 3 independent experiments. ^#^ *p* < 0.05, ^##^ *p* < 0.01 and ^###^ *p* < 0.001 vs. basal; * *p* < 0.05 and *** *p* < 0.001 vs. cytokines.

The effects of PS5 compound on the production of superoxide anion were analyzed in two cell types mostly involved in atherosclerotic disease: murine VSMCs (primary culture) and macrophages (RAW 264.7 cell line). It is well known that macrophages are the major immune cell population in the arterial plaques [51], while VSMCs contribute to vessel wall inflammation and lipoprotein retention, as well as to the formation of the fibrous cap responsible for the plaque stability [52]. Exploiting the ability of DHE probe to provide fluorescent signals upon the interaction with radical species, in the limits of a semiquantitative assay for the detection of the whole ROS content that changes continuously in a cell [53], in Figure 2 the confocal microscopy images of both cell types are reported. They clearly show that PS5 treatment caused a significant decrease in signal due to intracellular O_2_^•−^ production induced by cytokine stimulation, indicating a net antioxidant effect in both cellular systems. As expected, pretreatment with the Negative Control (NC) peptide did not cause any effect on ROS generation by VSMCs and macrophages.

**Figure 3:**
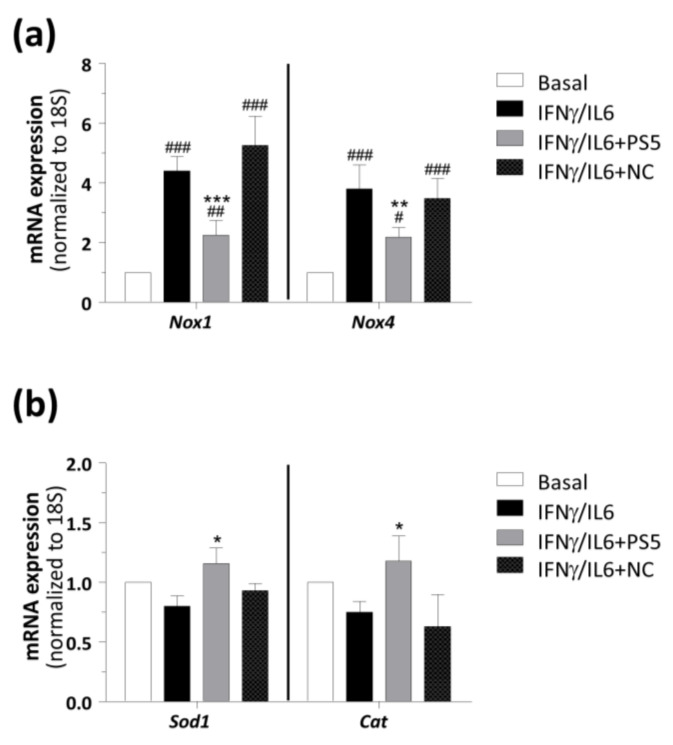
Real-time PCR analysis of STAT-dependent redox genes in VSMCs. The mRNA expression levels of (**a**) pro-oxidant (*Nox1* and *Nox4*) and (**b**) antioxidant (*Sod1* and *Cat*) enzymes were normalized to 18S and expressed as fold increases under basal condition. Results are presented as the mean ± SEM of *n* = 4–5 experiments (^#^ *p* < 0.05, ^##^ *p* < 0.01 and ^###^ *p* < 0.001 vs. basal; * *p* < 0.05, ** *p* < 0.01 and *** *p* < 0.001 vs. cytokines).

In this process, the generation of O_2_^•−^ radicals is regulated by NOX enzymes [54]. Thus, to explore in further detail the observed antioxidant effect of PS5, its ability to decrease the expression of main NOX isoforms in vascular cells was investigated. Interestingly, PS5 strongly reduced the gene expression of both *Nox1* and *Nox4* induced by cytokines, whereas NC was ineffective (Figure 3a). Moreover, to examine the role of PS5 compound in the improvement of antioxidant response, the gene expression of *Sod1* and *Cat* were also determined. These two antioxidant enzymes are able to reduce redundant ROS and are linked with the cellular anti-inflammatory response [55]. Real-time PCR analysis (Figure 3b) showed that the pretreatment of cells with PS5, but not with NC peptide, increased the expression of both antioxidant genes, probably as a compensatory mechanism to reduce oxidative stress induced by IFNγ/IL6.

### 3.2. Biodistribution and In Vivo Effects of PS5 Compound

#### 3.2.1. Atheroma Plaque Development

**Figure 4:**
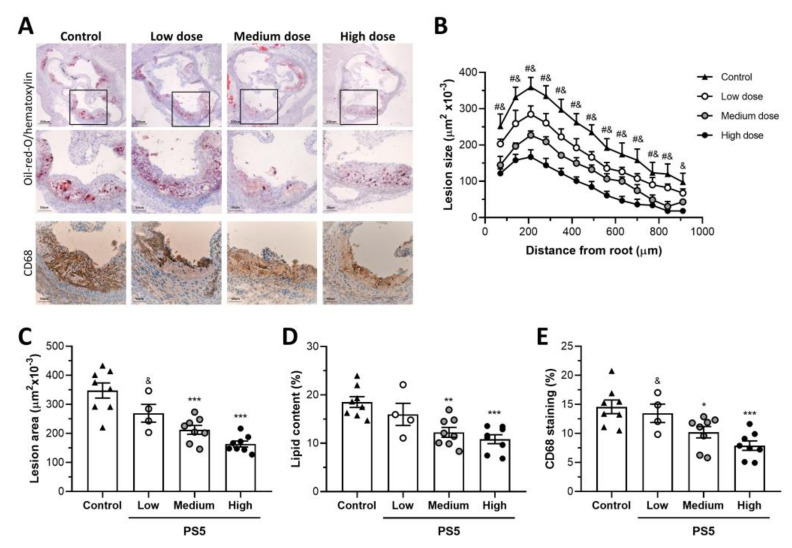
Effects of PS5 administration on atheroma plaques. (**A**) Representative images of Oil-red-O/hematoxylin staining and CD68 immunostaining (original magnifications, x40 and x100) in aortic sections of ApoE KO mice untreated (control, *n* = 8) and treated with low (19 µg/mouse, *n* = 4), medium (38 µg/mouse, *n* = 8) and high (79 µg/mouse, *n* = 8) doses of PS5 peptide. (**B**) Quantification of the extent of atherosclerotic lesions within the aorta. (**C**) Average of individual maximal lesion area in each group. Quantitative analysis of (**D**) lipid content and (**E**) CD68+ macrophages in atheroma plaques (calculated as % positive staining per lesion area). Results are presented as individual data points and the mean ± SEM. * *p* < 0.05, ** *p* < 0.01 and *** *p* < 0.001 vs. control; ^#^ *p* < 0.05 vs. medium dose; and *p* < 0.05 vs. high dose.

To further assess the atheroprotective properties of PS5, its effects were investigated in vivo using a well-characterized experimental model of atherosclerosis, the ApoE KO mouse under a high-fat diet. For biodistribution experiments, cell-permeable PS5 peptide conjugated to the fluorophore (TAMRA-PS5) was intraperitoneally injected into ApoE KO mice at 6 and 24 h before in vivo/ex vivo imaging analysis. As reported in Figure S1a,b, PS5 was mainly localized in the liver and showed a clearance through the renal system at 24 h postinjection. Furthermore, it was verified that PS5 effectively reached the target tissues (i.e., aorta) of ApoE KO mice. Confocal microscopy on mouse aortic sections (Figure S1c,d) revealed a cellular accumulation of labeled peptide in the plaque interior usually containing macrophages and VSMCs, but not in endothelial layer, and mostly at 6 hours after injection. Subsequently, the impact of PS5 peptide on the progression of atherosclerotic disease was investigated. For this, high-fat diet fed ApoE KO mice were treated for 8 weeks with three different doses of PS5 (19, 39 and 78 µg/mouse) referred to as low, medium and high. No differences in body weight were observed among the experimental groups (Delta body weight, g: control, 5.6 ± 0.9; low-dose, 5.3 ± 0.3; medium-dose, 5.8 ± 0.5; high-dose, 5.5 ± 0.9; *p* > 0.05 for all), as also reported in Figure S2. In Figure 4a, representative images of aortic root cross-sections stained with Oil-red-O, used to highlight in red lipidic aggregates are reported. Compared with untreated controls, PS5 treatment resulted in a dose-dependent reduction in atherosclerotic lesions. Quantification data revealed that both medium and high doses of PS5 significantly decreased the extension and average lesion size of atheroma plaques (% vs. control: 61 ± 4 and 47 ± 3, respectively; *p* < 0.0001) (Figure 4b,c) and also reduced the neutral lipid content (Figure 4d). The accumulation of monocytes/macrophages (CD68 immunohistochemistry, Figure 4a, within the atherosclerotic plaques was also reduced by PS5 treatment, with a significant effect at medium and high doses (% vs. control: 70 ± 7 and 54 ± 6, respectively; *p* < 0.002).

#### 3.2.2. JAK/STAT Regulated and Oxidative Genes in Aortic Samples

**Figure 5:**
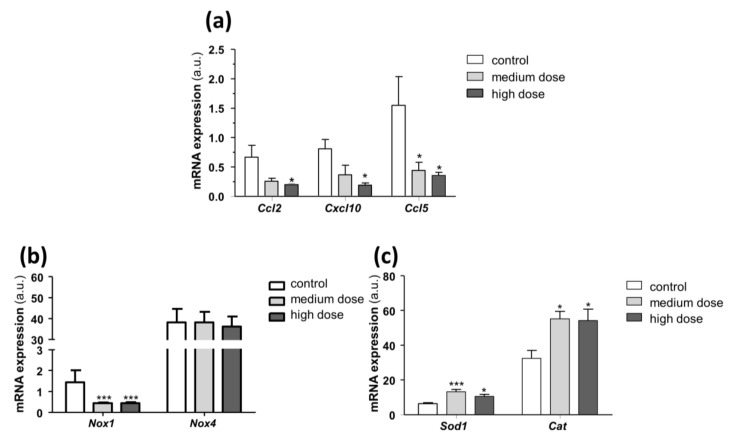
In vivo effects of PS5 on pro-inflammatory and redox-related genes. Real-time PCR analysis in aortic samples from ApoE KO mice untreated (control) and treated with medium- and high-dose PS5 peptide (38 and 79 µg/mouse, respectively). The mRNA expression levels of (**a**) *Ccl2, Cxcl10* and *Ccl5* chemokines, (**b**) *Nox1* and *Nox4* pro-oxidant enzymes and (**c**) *Sod1* and *Cat* antioxidant enzymes, were normalized to 18S and expressed as arbitrary units (a.u.). Results are presented as the mean ± SEM of *n* = 6–7 animals per group. * *p* < 0.05 and *** *p* < 0.001 vs. control.

To deepen the specificity of PS5 action as mimetic of SOCS1 in vivo, we investigated the effects on JAK/STAT regulated genes. Real-time PCR analysis of aortic samples from PS5-treated mice (Figure 5a) outlined a reduced gene expression of *Ccl2*, *Ccl5* and *Cxcl10* chemokines in mice treated with medium and high doses of PS5 when compared with untreated controls. Then, we also speculated on the antioxidant potential of PS5 in vivo. Indeed, the downregulation of superoxide-generating enzyme *Nox1* (Figure 5b) and the increased expression of the antioxidant enzymes *Sod1* and *Cat* (Figure 5c) confirmed the negative regulation of oxidative processes caused by PS5 therapy. Concerning Nox4 expression, we did not observe significant changes after PS5 administration (Figure 5b), which could be consistent with the protective role of Nox4 against atherosclerosis in murine models [56].

## 4. Conclusions

This study outlines the potentialities of a SOCS1-based therapy to limit the progression of atherosclerotic disease. The uncontrolled production of ROS is directly linked to vascular damage [57] and NOX proteins to ROS generation in the cardiovascular system [58]: in mice, NOX1 and NOX4 isoforms are largely implicated in diabetic disease and atherosclerosis, and their deletion can significantly attenuate these pathologies [56]. Conversely, in ApoE KO mice, the lack of efficient antioxidant systems increases atherosclerotic lesions and elevates mitochondrial ROS indicating a role for mitochondrial ROS in atherogenesis [59]. VSMCs can also be sources of ROS promoting oxidative stress [60] and various stimuli, including increased cyclic stretch, which can promote oxidative stress in VSMCs [61]. Activation of oxidative stress and inflammation occurs via receptors leading to changes in the balance between vasodilators and vasoconstrictors affecting vascular tone and ultimately leading to vascular dysfunction [60,62]. In the vascular wall, oxidative stress is counteracted by several antioxidant enzyme systems including SOD1 and CAT; indeed, in ApoE KO mice, the overexpression of both genes reduced atherosclerosis [63]. Recently, SOCS1 and SOCS3 were found in murine and human atherosclerotic lesions [25], and their anti-inflammatory and atheroprotective roles were outlined in vascular cells as endothelial and VSMCs: in them, SOCS1 gene delivery impaired pro-inflammatory gene expression, cell migration and proliferation [25]. In vivo, SOCS1^−/−^ triple-KO mice showed the total absence of atherosclerotic plaque formation, confirming the atheroprotective role of SOCS1 protein [64]. Herein, the effects of PS5, a peptidomimetic of SOCS1, were investigated in a cardiovascular disease model focusing on its antioxidant and atheroprotective properties. In vitro assays in VSMCs and macrophages revealed that PS5 inhibited ROS production, impaired vascular expression of NOX isoforms and prevented cell migration and proliferation without affecting cell viability. On the other hand, PS5 activated antioxidant defense mechanisms, as highlighted by the increased expression of antioxidant enzymes. In addition, PS5 treatment caused a significant decrease in fluorescent signal in the DHE experiment due to the intracellular production of O_2_^•−^ induced by proinflammatory cytokines (IFNγ/IL6), thus confirming its antioxidant power. In vivo, PS5 administration to atherosclerotic mice dose-dependently significantly decreased the size and extension of atheroma plaques, the intraplaque lipid content and the accumulation of monocytes/macrophages. Finally, the analysis of aortic samples from treated mice confirmed the anti-inflammatory and antioxidant effects of PS5, as evidenced by the downregulation of chemokines (*Ccl2*, *Ccl5* and *Cxcl10*) and superoxide-generating enzyme (*Nox1*) and the concomitant upregulation of antioxidant genes (*Sod1* and *Cat*). Our study demonstrates that PS5 therapy negatively regulates the oxidative processes acting as a potent antioxidant. Overall, data indicate that compounds mimicking SOCS1 could be valuable therapeutics in atherosclerosis.

## Figures and Tables

**Table 1:** Primers used in RT-PCR.

Gen Taqman	Primer Code	Gen Taqman		Primer Code
***Ccl2***	Mm00441242_m1	***Sod1***		Mm01344233_g1
***Ccl5***	Mm01302428_m1	***Catalasa***		Mm00437992_m1
***Cxcl10***	Mm00445235_m1	***18S***		4310893E
**Gen SYBR Green**	**Forward sequence (5’-3’)**		**Reverse sequence (5’-3’)**	
***Nox1***	CCAACAGGCCATGGATGGAT		CACTCCAGTAAGCCAGCAA	
***Nox4***	CCCTCCTGGCTGCATTAGTC		AACCCTCGAGGCAAAGATCC	
***18S***	CCGTCGTAGTTCCGACCATAA		CAGCTTTGCAACCATACTCCC	

## Supplementary Materials

The following are available online at https://www.mdpi.com/2076-3921/9/8/754/s1. Figure S1: biodistribution of PS5 peptide in ApoE KO mice. Figure S2: biodistribution of PS5 peptide in ApoE KO mice. (a) In vivo and (b) ex vivo images showing tissue biodistribution of TAMRA-labeled PS5 peptide at 6 and 24 h postinjection. Abbreviations: H, heart; L, liver; S, spleen; K, Kidney. Microscopic distribution of PS5 in mouse atherosclerotic plaque at (c) 6 h and (d) 24 h postinjection. Red, TAMRA-PS5; blue, DAPI. Dashed line: plaque area; arrows: positive cells.
