# Supplementary material for: Antioxidant Effects of PS5, a Peptidomimetic of Suppressor of Cytokine Signaling 1, in Experimental Atherosclerosis
Source: Antioxidants (Basel). 2020 Aug 14;9(8):754. doi: 10.3390/antiox9080754 (PMC7465353; doi:10.3390/antiox9080754)
Supplement: Supplementary file 1 [file antioxidants-09-00754-s001.pdf]

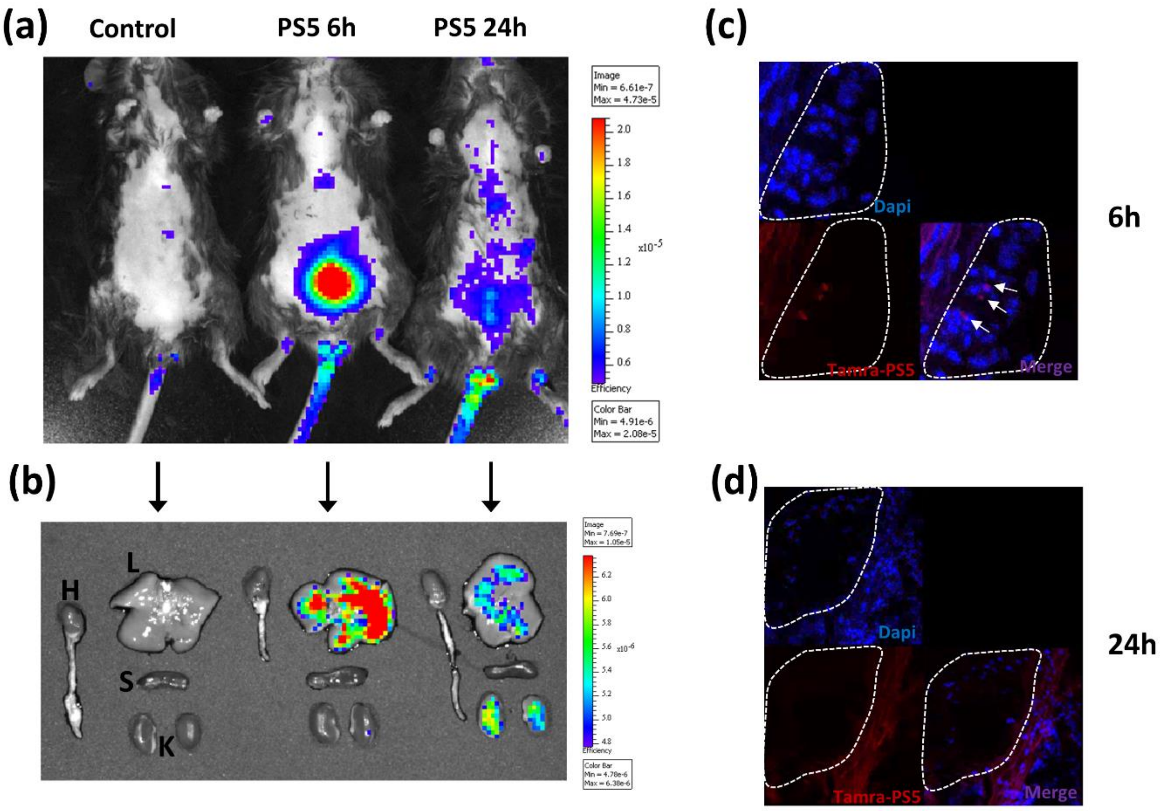

Figure S1. Biodistribution of PS5 peptide in ApoE KO mice. (**a**) *In vivo* and **(b)** *ex vivo* images showing tissue biodistribution of TAMRA-labeled PS5 peptide at 6 and 24h post-injection. Abbreviations: H, heart; L, liver; S, spleen; K, Kidney. Microscopic distribution of PS5 in mouse *atherosclerotic plaque* at **(c)** 6h and **(d)** 24h post-injection. Red, TAMRA-PS5; blue, DAPI. *Dashed line: plaque area, arrows: positive cells*

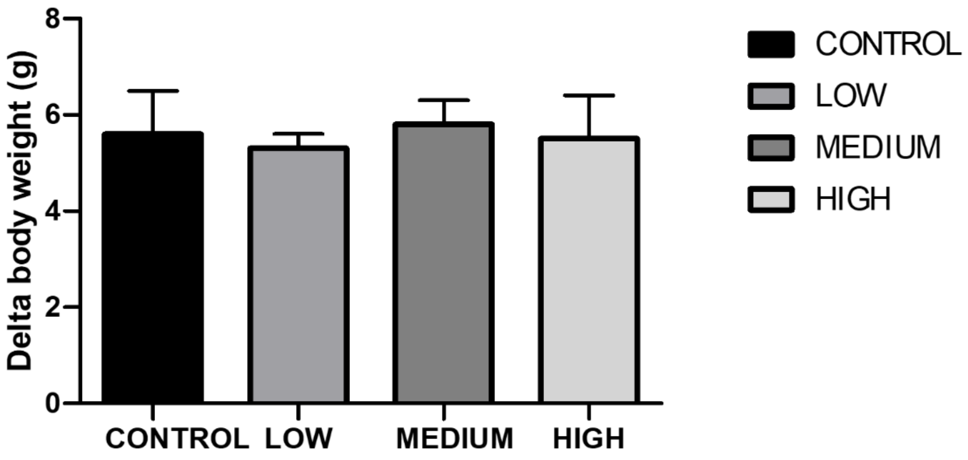

Figure S2. Averaged values of differences of body weights among mice groups evaluated weekly for 8 weeks
